# Tranexamic acid protects against implant-associated infection by reducing biofilm formation

**DOI:** 10.1038/s41598-022-08948-w

**Published:** 2022-03-22

**Authors:** Jiahao Wang, Zhen Zhang, Jingyi Li, Biying Huang, Zichao Jiang, Yixiao Pan, Tailai He, Yihe Hu, Long Wang

**Affiliations:** 1 Department of Orthopedics, Xiangya Hospital, Central South University, Changsha, China; 2 Department of Orthopedics, First Affiliated Hospital, School of Medicine, Zhejiang University, No.79 Qingchun Road, Hangzhou, 310003 Zhejiang China; 3 Hunan Engineering Research Center of Biomedical Metal and Ceramic Implants, Xiangya Hospital, Central South University, Changsha, China; 4 National Clinical Research Center for Geriatric Disorders, Xiangya Hospital, Central South University, Changsha, China; 5 Hunan Key Laboratory of Aging Biology, Xiangya Hospital, Central South University, 87 Xiangya Road, Kaifu District, Changsha City, 410008 Hunan China; 6 Department of Ultrasound Diagnosis, Second Xiangya Hospital, Central South University, Changsha, China

**Keywords:** Biochemistry, Microbiology, Medical research

## Abstract

Perioperative administration of tranexamic acid (TXA) is thought to be related to decreased postoperative implant-associated infection rates; however, the relationship remains unclear. We explored the inhibitory effect of TXA on infection both in vitro and in vivo. We investigated biofilm formation after TXA administration through different detection methods, all of which showed that TXA reduces biofilm formation in vitro and was further proven to be associated with decreased protein and polysaccharide contents in biofilms. We observed decreased biofilm on implants and decreased bacteria in the infection area with strengthened neutrophil accumulation in the mouse implant-associated infection model. Our results suggest that TXA protects against implant-associated infection by reducing biofilm formation in infected tissues.

## Introduction

Orthopaedic implants are routinely used during orthopaedic surgery, mainly including joint prostheses, orthopaedic plates, screws, and Kirschner wires. These implants provide mechanical support and motor functions and optimize skeletal function and alignment under physiological loading^1^. However, with the increasing use of such implants, implant-associated infection has become a challenge for surgeons. It is estimated that the infection rate ranges between 1 and 2% every year after knee or hip arthroplasties in the UK and USA and has continued to increase in recent years^2^. The reason for the infection is rooted in pathogens settling at the surgical sites or migrating from other places through blood circulation. Bacteria attach to the surface of implants and develop into biofilms. Biofilms have been proven to protect bacteria from bactericidal effects and play an important role in the formation of drug resistance^3^, which leads to difficulty in treating implant-associated infections in the clinic. Therefore, to solve the problem described above, it is important to focus on an effective therapy for biofilms formed on the implant surface.

Biofilms are a community of microorganisms in which bacterial cells are embedded in a matrix they produce, called extracellular polymeric substances (EPSs), which mainly consist of polysaccharides, proteins, lipids, and extracellular DNA^4^. Biofilms create a multifunctional and protective microenvironment in which microorganisms can interact socially and in which tolerant cells can survive for a long time, escape from changes in living conditions, and kill the immune system in the external environment. Therefore, the inhibition and removal of biofilms are essential for infection treatment^5^.

Tranexamic acid (TXA) is an antifibrinolytic that is routinely used as an effective prophylactic; it can prevent acute haemorrhage during orthopaedic surgeries. In 2010, a randomized controlled trial reported that TXA safely reduced the risk of death in bleeding trauma patients. All-cause mortality was significantly reduced after TXA treatment compared with that in a placebo group^6^. Since then, TXA has become widely adopted during surgeries, including joint surgeries^7^. Recently, some studies have reported that the use of TXA decreases periprosthetic joint infection^8–10^. A U.S. study including 914,990 total joint arthroplasty patients reported that the administration of TXA during surgery could result in significantly decreased odds of periprosthetic joint infection (PJI) within the first 90 days^8^. Another study investigated the infection rates of 6340 patients, including 3683 with TXA treatment and 2657 without TXA treatment, after total joint arthroplasty. The research showed that patients receiving TXA had significantly lower odds of infection^10^. However, the relationship between TXA administration and postoperative infection remains unclear. The mechanism behind the reported inhibitory effect of tigecycline on infection is still unknown. The reduction in peri-operative bleeding was thought to effectively contribute to lower postoperative infection rates^11^. A study explored the relationship between TXA and biofilms based on a mouse model of *Staphylococcus aureus* knee prosthesis infection. It was reported that TXA potentially promotes biofilm formation on the surface of articular implants in the prosthetic joint^12^. However, the study did not present detailed biofilm inhibition results of the in vivo test. The biofilm formation results of the in vivo test and the in vitro test were contradictory, and there was no reasonable explanation. There is still an urgent need for a study exploring the effect of TXA on biofilms and its mechanism.

In this study, the first aim was to elucidate the relationship between TXA administration and postoperative infection. The second aim was to investigate the relationship between TXA administration and biofilm formation, both in vitro and in vivo. The third aim was to clarify the new role and guide the application of tranexamic acid in joint surgeries. We hope to offer effective information about the utility of TXA in the prevention of implant-associated infections, as well as preventing blood loss.

## Results

### Successful construction of the GFP-transgenic *S. aureus* USA300 strain

**Figure 1:**
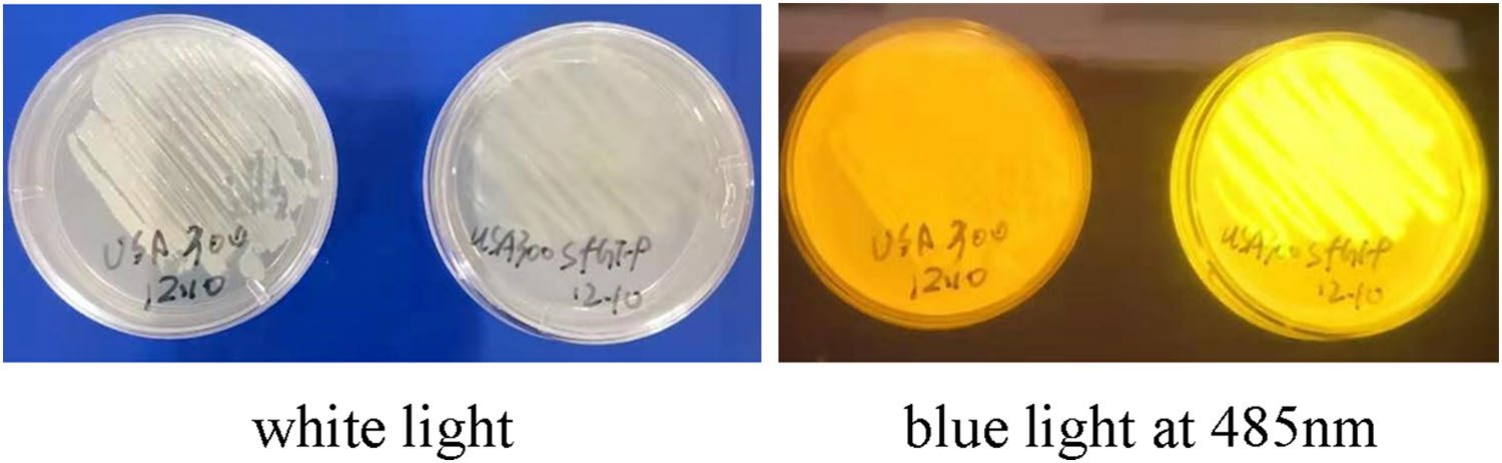
Original *USA300* strain (left plate) and GFP-transgenic *USA300* strain (right plate) under white light (left picture) and blue light at 485 nm (right picture).

To visualize the bacterial strains for morphological observation and biofilm detection, we transformed the GFP-expressing plasmid pRN-sfGFP into *S. aureus USA300*. As shown in Fig. 1, neither the original strain nor the transgenic strain exhibited fluorescence under white light, but the transfected bacteria could emit fluorescence at an excitation wavelength of 485 nm under blue light, while the original *USA300* strain could not, which indicated successful construction of the GFP-transgenic *S. aureus USA300* strain.

### Effects of TXA on biofilm formation in vitro

**Figure 2:**
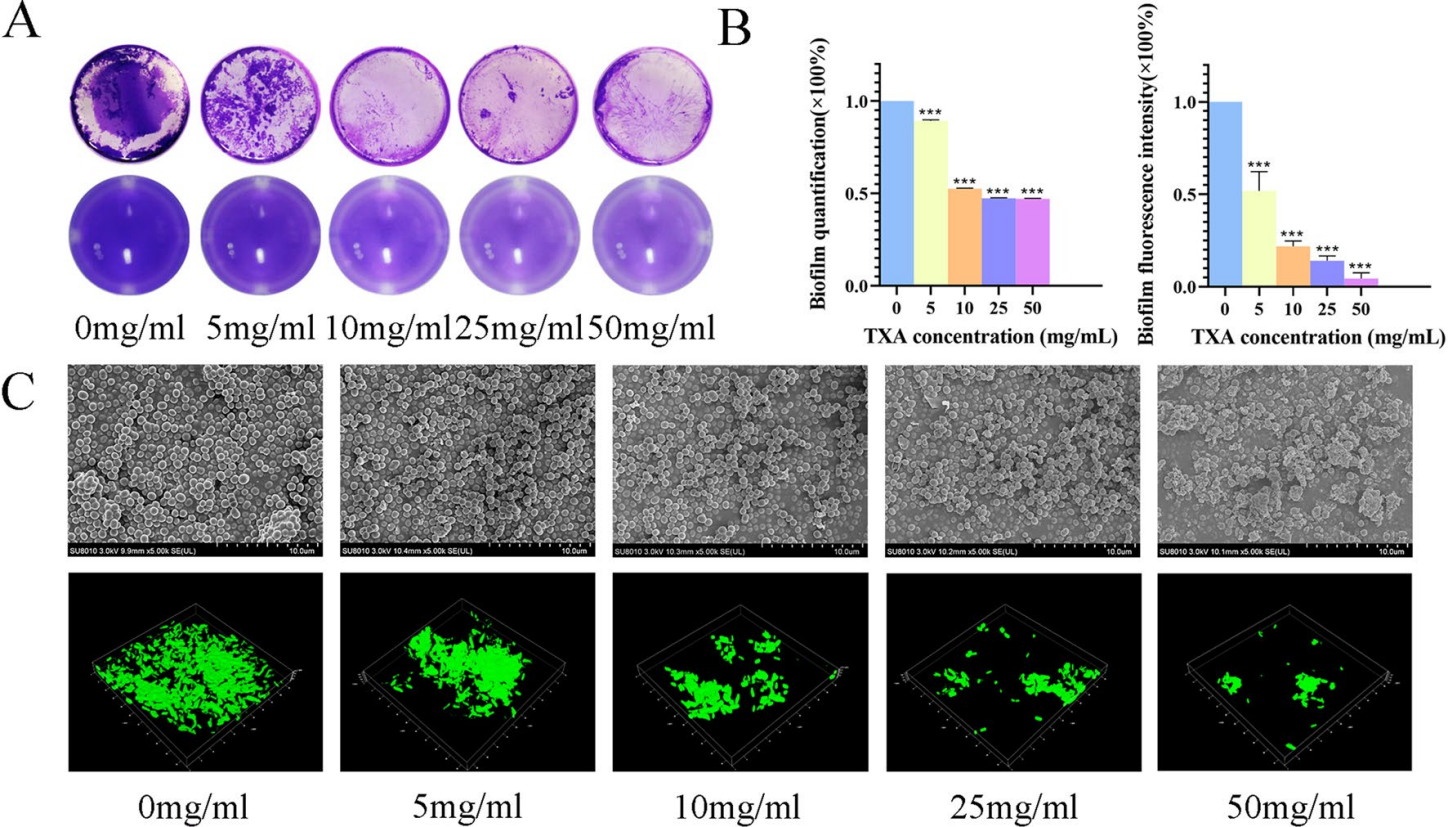
(**A**) The crystal violet staining results of biofilms after culturing with TXA at different concentrations for 36 h. (**B**) Biofilm quantification (left) of crystal staining and fluorescence intensity quantification (right) of CLSM after treatment with TXA at different concentrations for 36 h. (**C**) SEM (above) results of *USA300* biofilm and CLSM (below) results of *ATCC43300* biofilm after culturing with TXA at different concentrations for 36 h. The results in (**B**) are presented as the mean of three independent experiments of crystal staining and CLSM and analysed by one-way ANOVA (*p < 0.05 indicates statistical significance).

Figure 2A shows the results of the crystal violet staining test. The biofilm was analysed after incubating bacteria with TXA at different concentrations for 36 h. Compared with the control group, the TXA groups had less biofilm in a dose-dependent manner. The higher the concentration of TXA was, the less biofilm there was. Biofilms in the control group were stained purple, while the amount was smaller in the 5 mg/ml group and only small pieces were observed in the 10, 25, and 50 mg/ml groups. The biofilm was quantified by dissolving the dye in absolute alcohol, and the statistical results in Fig. 2B (left) show that TXA at concentrations of 10, 25, and 50 mg/ml inhibited biofilm formation with an inhibition rate greater than 50%.

SEM and CLSM were also used to further study the pharmacological inhibition effect by observing the changes in biofilm structures after TXA treatment. As shown in Fig. 2C (above), after incubation with TXA for 36 h, a biofilm was formed as a sheet of membrane composed of bacteria and secreted substances. In the 25 and 50 mg/ml groups, only small pieces of biofilm were observed, especially in the 50 mg/ml group. As Fig. 2C (below) shows, CLSM enabled clear observation of biofilms, which emitted green fluorescence after transfection of the GFP-expressing plasmid into the *USA300* strain. The biofilm was intact in the control group with strong green fluorescence. As the TXA concentration administered was increased, the biofilm gradually appeared as smaller pieces with decreased fluorescence intensity as well. Quantification of the fluorescence intensity as shown in Fig. 2B (right) revealed that TXA at concentrations from 10 to 50 mg/ml inhibited biofilm formation by approximately 80–90%, which was consistent with the crystal staining result.

Therefore, it could be concluded that TXA has an obvious inhibitory effect on biofilms.

### Effects of TXA on biofilm components

**Figure 3:**
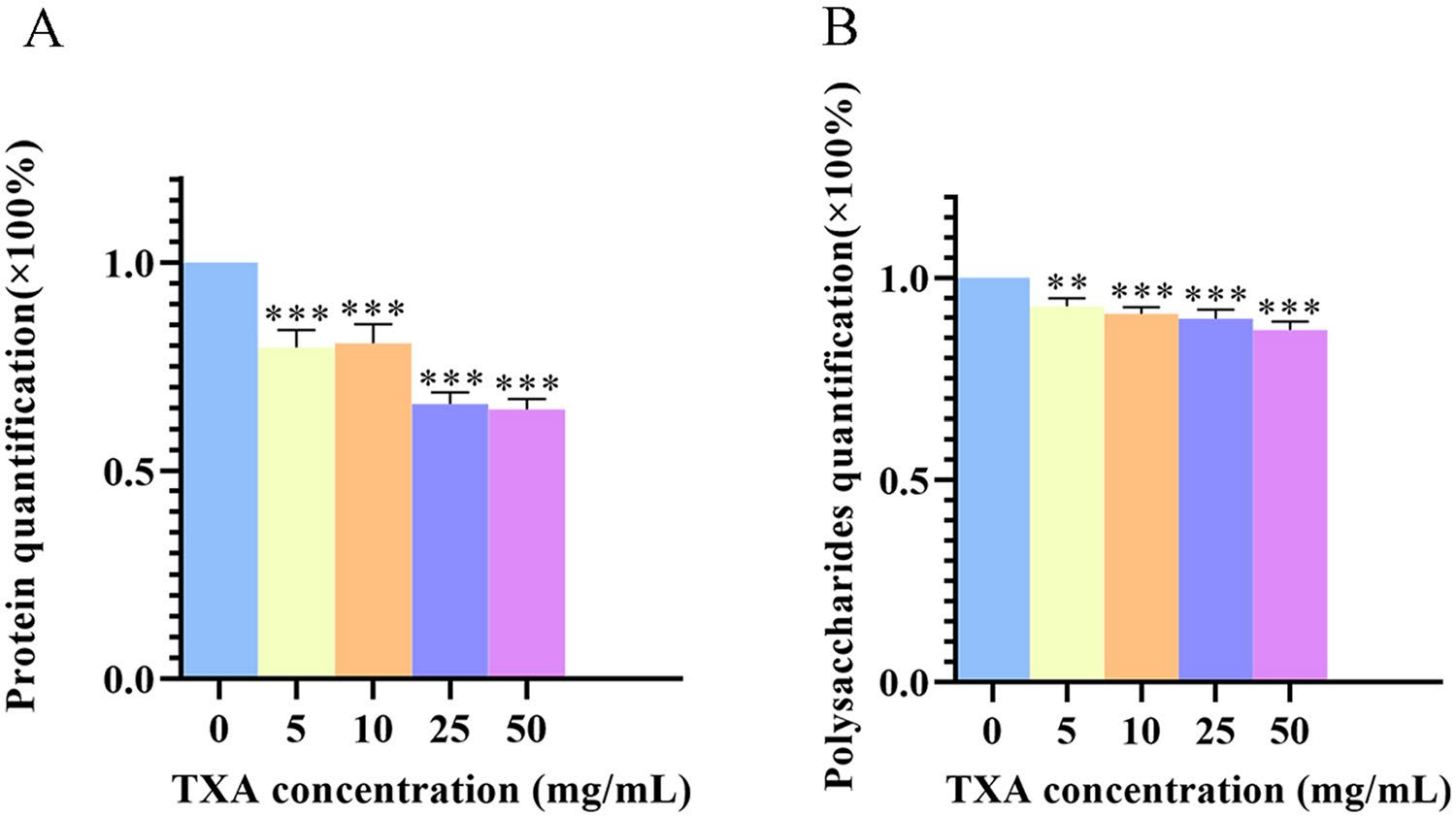
(**A**) Quantification of proteins and (**B**) polysaccharides in *ATCC43300* biofilm matrices after culturing with TXA at different concentrations for 36 h. The results are presented as the mean of three independent experiments and were analysed by one-way ANOVA (*p < 0.05 indicates statistical significance).

The above results showed that TXA performs dose-dependent inhibition of biofilm formation. Therefore, the changes in biofilm components were detected to obtain a better understanding of the mechanism of the inhibitory effect of TXA. It was previously reported that biofilms consisted mainly of polysaccharides and proteins, and *methicillin-resistant Staphylococcus aureus* (*MRSA*) had been observed to form biofilms mainly consisting of proteinaceous material^13^. Then, the proteins and polysaccharides in biofilm matrices were detected after treatment with TXA at different concentrations. TXA administration significantly reduced the protein components in biofilm matrices in a dose-dependent manner. The 5 and 10 mg/ml TXA treatments decreased biofilm formation by approximately 20%, while the 25 and 50 mg/ml TXA treatments achieved the highest inhibition rate of approximately 35%, as shown in Fig. 3A. The administration of TXA also decreased extracted polysaccharides compared with the control group in Fig. 3B, but there was no statistically significant difference among different concentrations, indicating that TXA inhibits biofilm formation mainly by suppressing protein components in biofilm matrices.

### Effects of TXA on biofilm formation in vivo: a mouse implant-associated infection model

**Figure 4:**
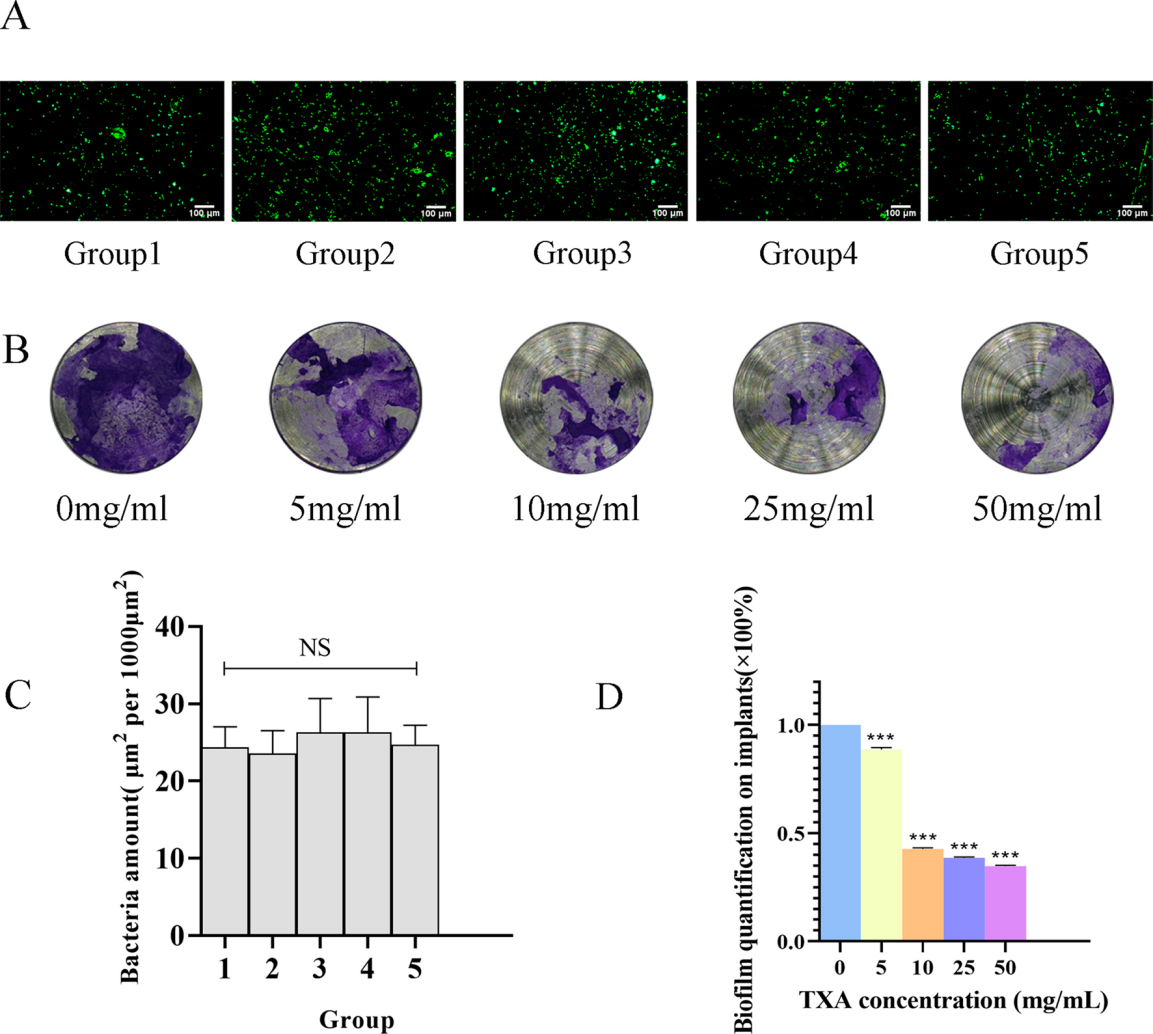
(**A**) Fluorescence observation (scale bar: 100 μm) and (**C**) quantification results of bacteria attached on disks 1, 2, 3, 4, and 5 after 24 h of culture. (**B**) Crystal violet staining results and (**D**) quantification of biofilms attached to implants after treatment with TXA at different concentrations for 3 days. The results were quantified as the mean of three independent experiments and analysed by one-way ANOVA (*p < 0.05 indicates statistical significance).

**Figure 5:**
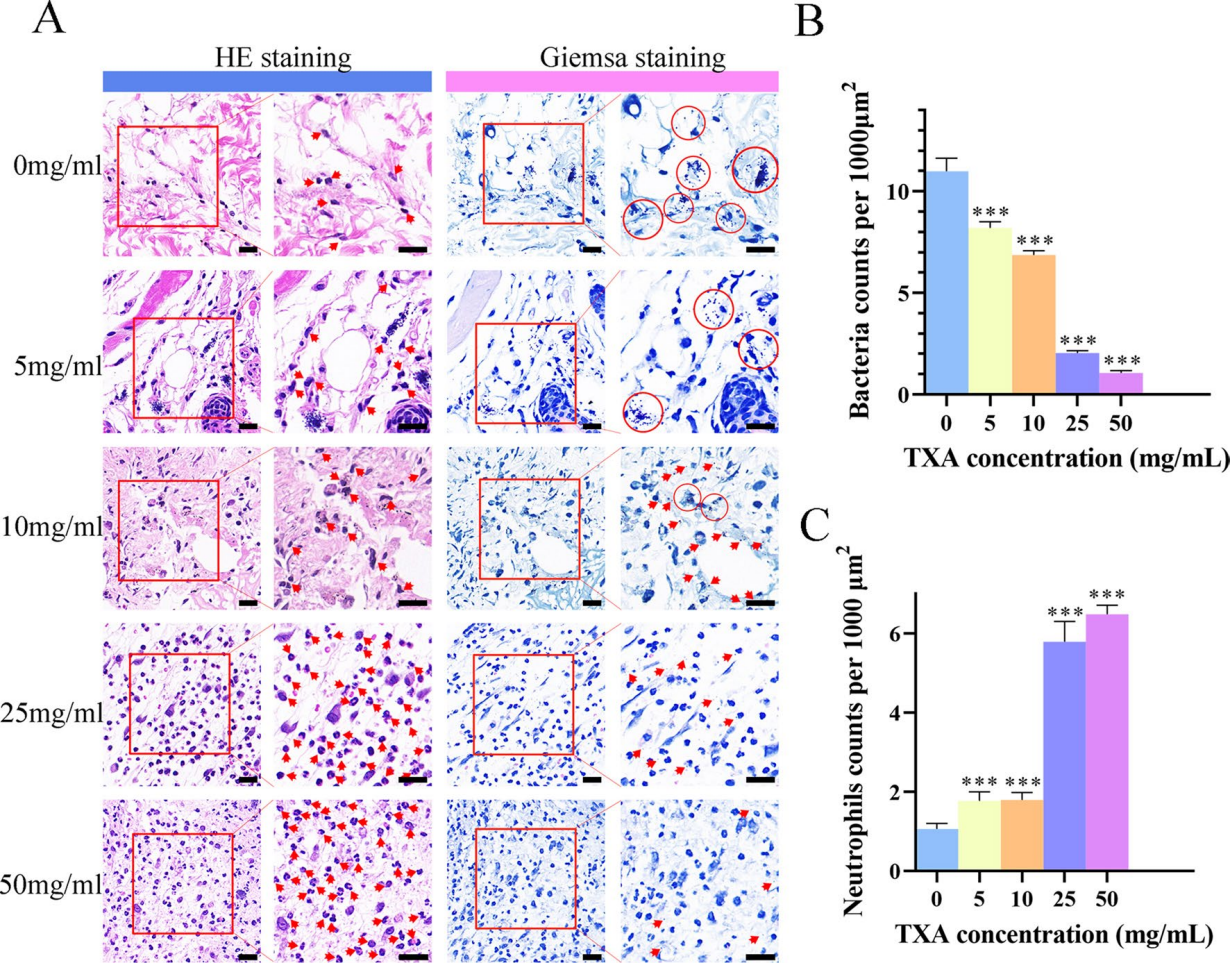
(**A**) HE and Giemsa staining of infection area tissue slices after administration of TXA at different concentrations 24 h after 3 injections. Red arrows represent neutrophils and bacteria in HE staining and Giemsa staining, respectively. Red circles represent the bacteria clumped together. Scale bar: 20 μm. (**B**, **C**) Quantification of bacteria and neutrophils in the infection area. The results were quantified as the mean of three independent experiments and analysed by one-way ANOVA (*p < 0.05 indicates statistical significance).

**Figure 6:**
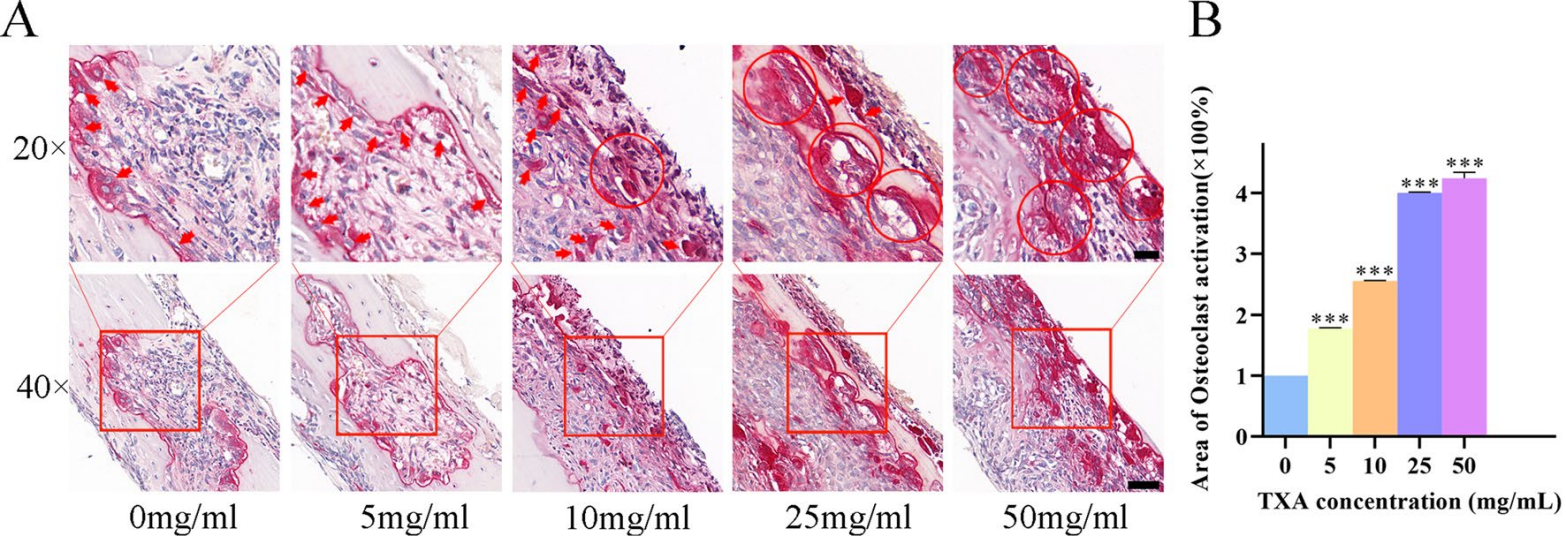
(**A**) Trap staining of skull slices after treatment with TXA at different concentrations 24 h after 3 injections. Scale bar: 50 μm (above) and 20 μm (below). (**B**) Quantification of osteoclast activation in skull slices. The results were quantified as the mean of three independent experiments and analysed by one-way ANOVA (*p < 0.05 indicates statistical significance).

Having demonstrated that TXA attenuated *S. aureus* biofilm formation mainly through the inhibition of proteins and polysaccharides, we finally investigated the biofilm-inhibiting effects through an in vivo test. The *S. aureus ATCC43300* strain successfully attached to titanium disks before implantation. The fluorescence results in Fig. 4A, C shows that the initial bacterial densities on different disks are equal. A mouse implant-associated infection model was successfully established by implanting titanium disks into the skull plates of mice, with equal amounts *MRSA* attached to the titanium disks. Disks were harvested 24 h after the third injection of TXA. Crystal violet staining was performed to investigate the antibiofilm efficiency of TXA on *MRSA* in vivo, as shown in Fig. 4B, D. The amount of biofilm formed on the implant surface decreased significantly in a dose-dependent manner with the administration of TXA. Biofilm was inhibited most obviously in the 50 mg/ml group, with an inhibition rate of more than 60%, as shown in Fig. 4D, indicating an effective antibiofilm performance of TXA at a high concentration. HE staining and Giemsa staining were also performed to investigate the inflammatory response and remaining bacteria around the implants, as shown in Fig. 5A, revealing an accumulation of neutrophils infiltrating into the infection area, and *S. aureus* could be observed as blue dots. Compared with the inflammatory cells in the control group, more infiltrated cells were found in the TXA-treated groups, and the cell number increased with increasing TXA concentration. Quantification was performed, and Fig. 5C shows that the numbers of infiltrated neutrophils in the 25 mg/ml and 50 mg/ml groups were both approximately 2 times greater than that in the control group. Furthermore, *S. aureus* could be seen accumulating within the infection area in the control, 5 mg/ml and 10 mg/ml groups, while the bacterial amount decreased as the TXA concentration increased. As shown in Fig. 5B, quantification revealed that the bacterial abundance in both the 25 mg/ml and 50 mg/ml groups decreased to less than 20% of that in the control group, indicating that the growth of *MRSA* was suppressed in vivo. As a previous study reported, some antibacterial drugs could improve the recruitment and engulfment of neutrophils in the infection area, which promoted the killing of *S. aureus*^14^. Therefore, the suppression of *MRSA* could contribute to the increased phagocytosis and clearance of *S. aureus* by the increasingly infiltrated inflammatory cells. As shown in Fig. 6, osteoclasts were stained red in the skull inflammatory area. It could be observed that with an increasing TXA concentration, osteoclasts around the implants in the TXA-treated groups were more activated than those in the control group, exhibiting a growing tendency, which suggested enhanced osteoclastic activity.

## Discussion

Implant-associated infection is a frightening complication that has occurred more frequently with the increasing usage of implants in orthopaedic surgeries in recent years. Nevertheless, few satisfactory therapies have been applied clinically to address the increasing rate of this infection. TXA is routinely used to prevent acute haemorrhage, especially in total joint arthroplasty. Recently, it was reported to have a potential downregulating effect on postoperative infection. Clinical researchers have found that it reduces the infection rate after total joint arthroplasty^9^, which allows us to recognize the utilization of tranexamic acid in orthopaedic surgeries in addition to haemostasis.

In addition, for patients undergoing total joint or knee arthroplasty, the dose of tranexamic acid varies from 10 to 50 mg/kg, with drug concentrations ranging from 10 to 50 mg/ml. The administration methods include intravenous, topical, and combined intravenous/topical methods^15,16^. There is still no absolute standard or recommendation for the use of TXA in clinical practice^17^.

Therefore, we performed this study to help clarify the relationship between tranexamic acid and infection and the effect of TXA on biofilms, which will also offer a new reference for the clinical application of tranexamic acid.

This study explored the effects of TXA on biofilm formation, revealing that TXA could reduce biofilm formation, especially in the high-concentration groups. The mechanism underlying the inhibition effect was determined: TXA reduced biofilm formation mainly by suppressing proteins and polysaccharide components. We finally investigated the effects of TXA on biofilms in vivo, presenting similar inhibition effects to the in vitro test, with an increased neutrophil response and decreased bacteria in the implants surrounding area. The groups receiving high intravenous doses exhibited the best biofilm inhibition performance in vivo.

We firstly focused on the effects of TXA on biofilm formation. The crystal violet staining test showed that biofilms could be inhibited by 10 mg/ml, 25 mg/ml, and 50 mg/ml TXA in vitro. Furthermore, clearer observation and quantification were obtained in this study through CLSM and SEM. These three TXA groups could also inhibit most biofilm formation, indicating that biofilm formation could be suppressed by high-concentration TXA treatment.

Since TXA exhibited an effective inhibitory effect on biofilm formation, there was a need to determine the mechanism behind this protective effect. Proteins and polysaccharides, two main components in *MRSA* biofilms, as previously reported^4^, were detected after TXA treatment. We discovered that TXA inhibited biofilm formation mainly by reducing the protein components, and 25 mg/ml and 50 mg/ml TXA also achieved better inhibitory performance.

Finally, we established a mouse implant-associated infection model for further exploration. Given that TXA is usually administered intravenously for 3 consecutive days after joint replacement surgery in the clinic, the mice received TXA intravenous injection after surgery. TXA is mainly eliminated through renal excretion, and approximately 90% of intravenously administered TXA was excreted within 24 h after injection^18^. Therefore, mice were injected once a day for 3 days, which precisely imitated the clinical usage of TXA, and euthanized 24 h after the third injection. Consequently, TXA exhibited a similar inhibition tendency on biofilm formation, especially in the 25 mg/ml and 50 mg/ml TXA groups, which confirmed that the protective effect of TXA could also be achieved in vivo. The ambient infiltrated neutrophils increased dose-dependently in the infection area, which was accompanied by decreased bacteria surrounding the infection area, revealing that TXA could positively influence neutrophil infiltration and consequently improve the clearing of pathogens. Osteoclast activation, which represented enhanced bone resorption activity, was also strengthened with increasing TXA concentration. However, there is no relevant research on the effect of TXA on osteoclasts in vivo. The relevant molecular mechanism remains unknown and needs further exploration.

Overall, 10 mg/ml, 25 mg/ml, and 50 mg/ml TXA inhibited biofilm formation, so we recommend 25 mg/ml and 50 mg/ml as the preferred TXA concentrations for topical use in joint surgeries. In addition, in the in vivo test, 25 mg/ml and 50 mg/ml TXA reduced the amounts of biofilm attached to implants and enhanced immune cell infiltration. The corresponding doses were 25 mg/mg and 50 mg/kg for 20-g mice. In a previous study, high-dose intravenously administered TXA was defined as a dose ≥ 20 mg/kg^16^. Therefore, high-dose intravenously administered TXA is more efficient in antibiofilm therapy. With less biofilm, antibiotics and immune molecules could enter the infection area more easily and efficiently, strengthening their anti-infection effectiveness. Considering all the results, we recommend the topical application of a TXA concentration of 25 mg/ml or more and an intravenously administered TXA dose of 25 mg/kg or more, which can effectively reduce the risk of infection by suppressing biofilms on the surface of the prosthesis, as well as reducing postoperative blood loss.

However, this study still has limitations. The specific mechanisms behind the antibiofilm effect of TXA remain unknown and deserve more research. Additional research is needed to examine shorter and longer time points and the molecular expression of biofilm-related genes. The ability of TXA to decrease the infection rate and improve prognosis requires more clinical investigation to evaluate and justify, which could be achieved through a large-scale randomized clinical trial.

Implant-associated infection is a serious complication affecting the postoperative prognosis of patients after orthopaedic surgeries, and biofilms contribute to the problem. This study showed that TXA, a widely used antifibrinolytic, could effectively inhibit bacterial biofilm formation both in vitro and in vivo, mainly through reducing the protein components in biofilms and accumulating neutrophils in the infection area. TXA at high concentrations and high doses is effective in suppressing biofilms, which contributes to the prevention of implant-associated infections and reduces blood loss. Even though the mechanisms behind the antibiofilm effect of TXA remain unknown and the clinical evaluation of the protective effects of TXA against implant-associated infection is limited, this study could contribute to a new understanding of the clinical use of TXA.

## Methods and materials

### Bacteria and animals

#### Bacterial strains and culture conditions

The *S. aureus* strains (*USA300*^19–21^ and *ATCC43300*^22,23^) used in the study were obtained from Xiangya Hospital of Central South University (Changsha, China). The *ATCC43300* strains were cultured in tryptic soy broth (TSB) culture medium, and the *USA300* strains were maintained in Luria Bertani (LB) broth containing 10 μg/ml chloramphenicol.

#### Mice

All the animal experiments reported followed the recommendations in the ARRIVE guidelines. Female BALB/C mice (6–8 weeks old with an average weight of 20 g) were purchased from HUNAN SJA Laboratory Animal Company and housed in the Department of Laboratory Animals, Central South University in China, for at least 7 days before use in this study. Experiments involving animals were all performed according to the guidelines of the Department of Laboratory Animals and approved by the Ethics Committee of Xiang Ya Hospital, Central South University in China (202108022).

### Effects of TXA on biofilm formation

#### Construction of green fluorescence-labelled *S. aureus* strain

Green fluorescence-labelled *S. aureus* was constructed to emit visible green fluorescence at an excitation wavelength of 485 nm by inducing a GFP-expressing plasmid into the bacteria. Briefly, the GFP-expressing plasmid pRN-sfGFP was first constructed with a constitutive promotor, which ensured the constitutive expression of fluorescent protein without inducer. After harvest of exponentially growing cultures of *S. aureus USA300*, the bacteria were washed with prechilled 0.5 M sucrose to be competent, after which the pRN-sfGFP plasmid was introduced by electroporation. Positive clones and recombinant strains were selected with PCR and cultured on LB plates containing 10 μg/ml chloramphenicol to observe the fluorescence at an excitation wavelength of 485 nm.

#### Crystal violet staining assays

The *S. aureus ATCC43300* strain was cultured in TSB broth at 37 °C overnight and suspended in fresh TSB broth containing 0.5% glucose (w/v) at a concentration of 5 × 107 CFU/ml. Then, 100 μl of bacterial suspension and 100 μl of TXA were added to a flat bottom polystyrene 96-well plate (NEST Biotechnology) with final TXA concentrations of 0, 5, 10, 25, and 50 mg/ml and incubated statically at 37 °C in a non-humidified incubator for 36 h to form biofilms. Thereafter, the wells were washed three times with PBS to remove untouched cells and then fixed with 4% paraformaldehyde for 10 min. After removing paraformaldehyde, the biofilm was stained with 0.1% (w/v) crystal violet staining solution for 30 min. The wells were then washed with running tap water until the water was clear. Finally, the biofilm was quantified by dissolving the dye in 100 μl of absolute alcohol and detecting the absorbance at OD570 nm. The experiment was repeated 3 times in 3 different weeks, and for each experiment, there were 3 replicate wells in each group. The experiment was conducted following a minimum information guideline for biofilm assessment^24^.

#### Observation of biofilms by confocal laser scanning microscopy (CLSM)

*S. aureus* strain *USA300* was cultured statically with TXA at 0, 5, 10, 25, and 50 mg/ml in confocal dishes at 37 °C for 36 h. Thereafter, the biofilm was harvested in confocal dishes. The biofilm was washed three times with PBS. Green fluorescence was emitted at an excitation wavelength of 488 nm. The biofilm was observed, and three-dimensional images of the biofilm were generated through a confocal laser-scanning microscope (CLSM, Zeiss LSM 510). The experiment was repeated 3 times, and in each experiment, there were 3 replicate samples in each group.

#### Morphological observation of biofilms by scanning electron microscopy (SEM)

Biofilms of the *S. aureus ATCC43300* strain were obtained following the method described above and fixed with 2.5% glutaraldehyde at 4 °C. After that, biofilms were dehydrated at progressively increasing ethanol concentrations, critical point dried, and coated with osmium. A scanning electron microscope (JEOL-7800F) was used to observe the samples. The experiment was repeated 3 times, and in each experiment, there were 3 replicate samples in each group.

### Effects of TXA on biofilm components

#### EPS extraction assay

Biofilms of the *S. aureus ATCC43300* strain were obtained with an initial concentration of 5 × 10^7^ CFU/ml with or without treatment with TXA after incubation for 36 h. EPS extraction was conducted according to previously described protocols^25^. Briefly, samples were suspended in 6 ml of phosphate-buffered saline (PBS). After pretreatment with ultrasound at 20 kHz and 40 W for 30 s and centrifugation at 2000×g and 4 °C for 15 min, soluble EPS was collected in the supernatant liquor. The sediments were resuspended in 2 ml of PBS, and 0.06 ml of 37% formamide was added to the suspension at 4 °C. After 1 h, the suspension was centrifuged at 5000×g and 4 °C for 15 min, and the supernatant was collected. Sediments were resuspended again with 8.5% sodium chloride (diluted in PBS). The suspension was treated with ultrasound at 20 kHz and 120 W for 3 × 2 min and centrifuged at 10,000×g for 15 min at 4 °C. Finally, all supernatant was sterilized through a 0.22-μm filter and stored at − 20 °C.

#### Quantitative detection of total polysaccharides in EPS

The phenol–sulfuric acid method was used to detect the polysaccharide components in the EPS. Briefly, 5% phenol and concentrated sulfuric acid were mixed in a volume ratio of 1:5 to prepare a developer solution. Then, 0.5-ml samples were added to 4.5 ml of developer solution and heated in boiling water for 30 min, and the absorbance was measured at OD490 nm. The experiment was repeated 3 times, and in each experiment, there were 3 replicate samples in each group.

#### Detection of protein in EPS

The protein component was detected by the BCA (Bicinchoninic Acid) Protein Assay Kit (New Cell & Molecular Biotech Co., Ltd) with bovine serum albumin as a standard. Briefly, the working fluid was configured by mixing BCA-A Solution and BCA-B Solution at a volume ratio of 50:1. Then, 200 μl of the working fluid was mixed with 20 μl samples in 96-well plates, and the absorbance was measured at OD562 nm after incubation at 37 °C for 30 min. The experiment was repeated 3 times, and in each experiment, there were 3 replicate samples in each group.

### Mouse implant-associated infection model and in vivo test

Thirty BALB/c mice, weighing 20 g apiece, were randomly divided into five groups at different TXA treatment concentrations: (1) 50 mg/ml; (2) 25 mg/ml; (3) 10 mg/ml; (4) 5 mg/ml; and (5) PBS. After anaesthesia with 1% pentobarbital sodium, injected intraperitoneally, the skull skin was carefully shaved, exposed, and cleansed with alcohol. A 4-mm incision was made in the middle area of the exposed skin of the skull. After peeling off the periosteum, a 5-mm titanium disk was implanted into the skull. Bacteria attached to the disks after incubation with 1 × 10^7^ CFU/ml *S. aureus ATCC43300* strain in fresh TSB broth containing 0.5% glucose for 24 h. Injections of different concentrations of TXA or PBS were given intravenously in a volume of 20 μl once a day. The corresponding doses were 5 mg/kg, 10 mg/kg, 25 mg/kg, and 50 mg/kg. Twenty-four hours after the third injection, the mice were humanely euthanized with an overdose of anaesthetics. Metal sheets were removed to detect the biofilm using crystal staining. Infected skin and skull bone were collected for H&E, Giemsa, and trap staining to analyse the effect of TXA on biofilm formation, inflammation, and osteolysis.

Five titanium disks, numbered 1, 2, 3, 4, and 5, were used to prove the equal bacterial density on the disks after culture in vitro. Briefly, titanium disks were cultured with 1 × 10^7^ CFU/ml *S. aureus ATCC43300* strain in fresh TSB broth containing 0.5% glucose for 24 h in 24-well plates. Then, the disks were washed 3 times with PBS and stained with a Calcein-AM/PI Double S7 stain kit (YEASEN, China) for 15 min at 37 °C. The bacteria were stained with green fluorescence and observed with a Leica fluorescence microscope (Leica MICROSYSTEMS). The bacterial numbers were counted with ImageJ software. The test was repeated 3 times on 3 different days in different 24-well plates.

### Statistical analysis

All results are shown as the mean values ± standard deviations after repeating at least three times. One-way ANOVA and Student's t test were adapted to analyse the data by using GraphPad Prism 5 software. *p < 0.05 was considered statistically significant.
